# Development of a Hyaluronic Acid-Based Nanocarrier Incorporating Doxorubicin and Cisplatin as a pH-Sensitive and CD44-Targeted Anti-Breast Cancer Drug Delivery System

**DOI:** 10.3389/fphar.2020.532457

**Published:** 2020-08-28

**Authors:** Tao Yu, Yongshuang Li, Xueyuan Gu, Qin Li

**Affiliations:** ^1^Center for Translational Medicine, the Fourth Affiliated Hospital of China Medical University, Shenyang, China; ^2^Department of General Surgery, the Fourth Affiliated Hospital of China Medical University, Shenyang, China; ^3^Centeral Laboratory, the Fourth Affiliated Hospital of China Medical University, Shenyang, China

**Keywords:** hyaluronic acid, doxorubicin, CD44, pH-sensitive, breast cancer chemotherapy

## Abstract

Tumor-targeting nanomaterial-based chemotherapeutic drug delivery systems have been shown to represent an efficacious approach for the treatment of cancer because of their stability in blood circulation and predictable delivery patterns, enhanced tumor-selective drug accumulation, and decreased toxicity to normal tissues. The cell-surface transmembrane glycoprotein CD44 binds to the extracellular domain of hyaluronic acid (HA), and is overexpressed in breast, ovarian, lung, and stomach cancer. In this study, an HA-based nano-carrier incorporating doxorubicin (DOX) and cisplatin (CDDP) was synthesized as a CD44-targeting anti-cancer drug delivery system, and its tumor inhibition effects against CD44^+^ breast cancer cells were evaluated *in vitro* and *in vivo*. These dual drug-loaded HA micelles (HA-DOX-CDDP) exhibited significantly enhanced drug release under acidic conditions, and showed higher cellular uptake and stronger cellular growth inhibition than free drugs against 4T1 (CD44^+^) breast cancer cells. In contrast, no significant differences in growth inhibition and cellular uptake were observed between HA-DOX-CDDP and free drugs in NIH-3T3 (CD44^-^) control cells. Furthermore, HA-DOX-CDDP micelles exhibited stronger inhibitory effects and lower systemic toxicity than free drugs in a 4T1 mammary cancer-bearing mouse model, as determined using immunofluorescence and histological analyses. Therefore, HA-DOX-CDDP micelles represent a promising drug delivery system that exhibits acid-sensitive drug release, CD44-targeted delivery, and excellent biocompatibility and biodegradation. These properties resulted in excellent tumor accumulation and reduced adverse effects, indicating that HA-DOX-CDDP micelles have promising potential applications in chemotherapy for breast cancer.

## Introduction

Breast cancer is the most serious threat to female health globally because of the associated high morbidity, poor prognosis, and limited availability of effective therapies. Breast cancer chemotherapy typically involves the administration of a single anti-tumor drug, and side effects and drug resistance remain significant challenges. Combined and targeted anticancer therapies have replaced conventional medical treatments owing to their enhanced breast tumor-targeting and improved drug delivery. However, a majority of currently used drug carriers have limitations that restrict their clinical application, such as the lack of long-term safety, poor biocompatibility, high cost of manufacturing, and limited drug loading efficiency (Cheng et al., 2014; Feng et al., 2017; Freitas de Freitas et al., 2018). Therefore, the development of targeted anticancer drug delivery systems with low toxicity and good biocompatibility is critical to improve the efficacy of breast cancer chemotherapy.

Previous studies have shown that polymeric nanoparticles (NPs) can be surface-engineered to act as drug delivery vehicles to improve biocompatibility, increased cellular uptake, and specific tumor targeting, which may result in targeted drug delivery and reduced adverse effects (Bahrami et al., 2017; Mu et al., 2017; Parashar et al., 2018). Hyaluronic acid (HA), a major component of the extracellular matrix, is frequently used as a nanocarrier in the biomedical and cosmetic industries because of its excellent water-binding properties, biocompatibility, biodegradation, and receptor targeting (Papalia et al., 2017; Michaud, 2018; Wang, 2018). Previous studies show that HA can be used as tumor site-specific drug delivery vehicle towing to its high binding affinity for the CD44 receptor (a member of the cell adhesion protein family), which is over-expressed on the surface of various carcinoma cells, including breast cancer (Louhichi et al., 2018) and lung cancer (Mattheolabakis et al., 2015). In contrast, CD44 has been shown to be expressed at low levels on normal cells (Cortes-Dericks and Schmid, 2017; Li et al., 2017). Therefore, HA-modified nanoparticles or micelles are promising as potential carriers for CD44-targeted chemotherapeutic agents.

Treatments using combinations of drugs with different physico-chemical properties may have limitations such as unexpected drug release. Cisplatin (i.e. cis-diamminedichloroplatinum, CDDP), a representative chemotherapeutic drug for various types of cancers, has been extensively formulated into NPs as cross-linked micelles to increase tumor targeting ability through high delivery efficiency and enhanced permeability and retention (EPR) effect at low pH (Ganesh et al., 2013; Fan et al., 2015; Alam et al., 2017; Cai et al., 2017; Girma et al., 2018). The tumor microenvironment is generally acidic, whereas normal tissues are neutral in pH or slightly alkaline (Katheder et al., 2017; Zheng et al., 2020). Therefore, we hypothesized that the pH sensitivity of the present CDDP-crosslinked HA-modified antineoplastic nanoparticles may result in modified drug release behavior in tumor tissues, thereby leading to reduced side effects.

In the present study, HA-modified nanoparticles crosslinked with CDDP and loaded with doxorubicin (DOX) as the anticancer drug were developed as a tumor-targeting formulation for breast cancer chemotherapy. The physicochemical properties, antitumor efficacy, and mechanisms of action of the HA-DOX-CDDP micelles were investigated using CD44^-^ normal fibroblast cells (NIH-3T3) and CD44^+^ breast cancer cells (4T1) *in vitro*, and in mice bearing 4T1 breast tumors *in vivo*.

## Materials and Methods

### Materials

Hyaluronic acid (MW = 1.0 × 10^5^ Da) was purchased from Freda Biochem Co., Ltd (Shandong, China). Doxorubicin hydrochloride (DOX HCl) was purchased from Dalian Meilun Biotechnology Co., Ltd. (Purity 98%, Dalian, China). CDDP was purchased from Shandong Boyuan Pharmaceutical Co., Ltd. (Purity 99%, Jinan, China). Poly (ADP-ribose) polymerase (PARP) and survivin antibodies were purchased from Abcam (Cambridge, UK). Purified deionized water was prepared using a Milli-Q plus system (Millipore Co., Billerica, MA, USA).

### Cell Lines and Animal Models

Murine CD44^+^ mammary carcinoma cells (4T1) and normal CD44^-^ fibroblast cells (NIH-3T3) were purchased from American Type Culture Collection (ATCC, Rockefeller, Maryland, USA), and cultured at 37°C in DMEM supplemented with 10% FBS, penicillin (50 U/ml) and streptomycin (50 U/ml).

Female BALB/c mice (4–5 weeks old) were purchased from the Experimental Animal Center of Jilin University, and housed in a standard environment with access to normal chow diet until they reached a weight of 18–20 g. A tumor-bearing model was generated by inoculating 1.5 × 10^6^ 4T1 cells into the abdominal mammary glands of female BALB/c mice. All animal procedures were designed to minimize animal suffering and were approved by the Institutional Animal Care and Use Committee of Jilin University.

### Preparation of HA-DOX-CDDP Nanoparticles

Hyaluronic acid-DOX-CDDP nanoparticles were prepared according to previous studies (Zhang et al., 2018; Zhao et al., 2018; Wang et al., 2019). Hyaluronic acid and phosphate buffer (pH=7.4, 1:9 volume ratio) were blended in deionized water until the HA was completely dissolved. Following dissolution, the pH was adjusted to 7.0 using 0.05 M NaOH. An aqueous DOX solution (6.0mg/ml) was added dropwise into the polymer solution, then stirred overnight in the dark at room temperature. Next, a predetermined amount of CDDP was dissolved and added into the reaction mixture mentioned above, and then, incubated at 37°C for 72 h. Finally, HA-DOX-CDDP was obtained using a dialysis method with deionized water as the releasing medium for 10 h.

### Characterization of HA-DOX-CDDP Nanoparticles

Dynamic light scanning (DLS) (Wyatt Technology, Santa Barbara, CA, USA) and transmission electron microscopy (TEM) (JEM-1011, JEOL, Japan) were performed to determine HA-DOX-CDDP nanoparticle size and morphology, respectively. Five milliliters of drug-loaded nanoparticles (0.1 mg/ml) were added to a clean glass container and dissolved prior to DLS detection. Ten microliters of drug-loaded nanoparticles (at the same concentration of 0.1 mg/ml) were added to a copper wire and completely dried for TEM analysis.

### *In Vitro* Drug Release

To determine the release characteristics of HA-DOX-CDDP in PBS (pH 5.5, pH 6.8, and pH 7.4), 1.0 mg of freeze-dried HA-DOX-CDDP micelles was used to prepare release diluent (100.0 mg/ml). The micelles were sealed with 10.0 ml of diluted solution in a dialysis bag (MW cut-off 3,500 Da) and placed in 100.0 ml of release medium, and the solution containing the dialysis bag was shaken at 70 rpm at 37°C. At 0.5, 1, 2, 4, 8, 12, 24, and 48 h, 2.0 ml of release diluent was removed and replaced with fresh diluent. The amounts of released DOX and CDDP were determined using a fluorescence spectrophotometer (UV-1800, Shimadzu, Japan) (λex = 480 nm and λem = 590 nm) and an inductively coupled plasmae mass spctrometer (ICP-MS, Xseries II, Thermoscientific, USA), respectively.

### *In Vitro* Cellular Uptake

Cellular uptake and intracellular DOX release of HA-DOX-CDDP micelles, free DOX, and free CDDP in 4T1 and 3T3 cells were assessed using flow cytometry (FCM) (Beckman, California, USA). Cells were seeded in 6-well plates at a density of 2×10^5^ cells per well, then incubated for 12 h at 37°C. The original culture medium was replaced with medium containing free DOX+CDDP or HA-DOX-CDDP at a final DOX HCl concentration of 10.0μmg/ml. In the HA-pretreated groups, cells were incubated with HA micelles for 1 h prior to treatment. Following pretreatment, the medium was replaced with medium containing HA-DOX-CDDP at the same concentration. After 6 h of incubation in a thermostatic incubator, the cells were harvested, washed in cold PBS, and centrifuged twice at 4°C for 5 min at 1,500 rpm. Finally, 500.0 μl of cell suspension was subjected to FCM analysis.

The cellular localization of DOX in 4T1 and 3T3 cells was determined using an LSM 780 confocal laser scanning microscope (CLSM) (Carl Zeiss, Jena, Germany). Cells (1.5 × 10^5^) were incubated on cover slips in 6-well plates for 12 h, and the medium was replaced with medium containing HA-DOX-CDDP micelles, or free DOX+CDDP in DMEM at a final DOX HCl concentration of 10.0 μg/ml. Hyaluronic acid pretreatment groups were pretreated with HA, and then incubated with HA-DOX-CDDP. After incubation for 6 h, cells were fixed in 4% (W/V) PBS-buffered formaldehyde for 30 min at 15–20°C. The cells were then stained with DAPI (blue) and Alexa 488 (green) at 37°C to visualize the cell nucleus and cytoskeleton, respectively.

### *In Vitro* Cytotoxicity Assay

The cytotoxicity of HA-DOX-CDDP was evaluated using the MTT assay. Briefly, 7 × 10^3^ 4T1 or NIH-3T3 cells per well were seeded in 96-well plates and incubated for 12 h. Two hundred microliters of culture medium was replaced with fresh medium containing free DOX+CDDP, HA-DOX-CDDP, or HA micelles at various concentrations, and each incubated for 24 or 48 h. Then, 20.0 μl of MTT (5.0 mg/ml) was added to each well, and the cells were incubated for 4 h at 37°C. Next, the medium was replaced with 150.0 μl of DMSO to dissolve the formazan crystals, and the cells were shaken for 10 min prior to analysis. Absorbance was measured at 490 nm using a microplate reader (Bio-Rad 680, Hercules, CA, USA). Cell viability was calculated using equation 1:

$$\text{Cell viability }(\%)=\frac{A_{\text{sample}}}{A_{\text{control}}}\times 100$$

In Eq. 1, *A*_sample_ and *A*_control_ represent the absorbances of sample and control wells, respectively.

### *In Vitro* Analysis of Morphology of Multicellular Spheroids in 3D Suspension Cultures

A 3D cell suspension cultures model was established using HDP 1096 Perfecta 3D^®^96-well hanging drop plates (3D Biomatrix, USA) as described in a previous study (Xiao et al., 2018). Briefly, suspensions of 4T1 and NIH-3T3 cells were added into agarose solution preprocessed 96-well plates at 2.5 × 10^4^ cells per well, and the cells were incubated at 37°C. After 5 days, multicellular spheroids were randomly divided into HA (control), free DOX+CDDP, and HA-DOX-CDDP groups with a final DOX HCl concentration of 10.0 μg/ml. Morphologic changes of the multicellular spheroids were visualized using CLSM after treatment for 24 h.

### *In Vivo* Fluorescence Imaging of DOX Biodistribution

The biodistribution of DOX in major internal organs and tumor tissues following intravenous injections was assessed using fluorescence imaging *in vivo*. When tumors grew to 60–80 mm^3^, the 4T1 tumor-bearing mice were injected with 0.1% normal saline (NS) as a control, HA-DOX-CDDP micelles, or DOX+CDDP *via* the lateral tail vein, at a DOX HCl dose of 5.0 mg/kg. Major organs (heart, liver, spleen, lung, and kidney) and tumors were excised 6 or 12 h post-injection, washed with NS three times, and analyzed for DOX-related fluorescence using an *in vivo* imaging system (Maestro 500 FL, Cambridge Research & Instrumentation, Inc., USA). A 150 W halogen lamp and a 450–500 nm excitation filter were used for DOX fluorescence analysis. Averaged signals with autofluorescence excluded were quantitatively analyzed using Maestro™2.4 software. Tumor volume (V) was calculated as 0.5 × length × width × height.

### *In Vivo* Antitumor Assay

*In vivo* antitumor efficacies of free drugs and HA-DOX-CDDP micelle were evaluated using the 4T1-xenografted Balb/c mice. Similarly to the detections of tissue distributions, 0.1 ml of cell suspension containing 1.5 × 10^6^ 4T1 cells in NS was injected subcutaneously into the abdominal mammary gland of 4–5 weeks old mice weighing of 18–20 g. When the tumor volume increased to about 60–100 mm^3^, the mice were treated with NS, combination of free DOX and CDDP, or HA-DOX-CDDP micelle at a DOX concentration of 5.0 mg/kg^−1^ body weight by the tail-veil injections on 0, 5, 10, 15, and 20 days. In the course of treatment, tumor volumes and body weights were monitored every other day. Tumor volume (V) was calculated as 0.5 × length × width × height.

### Immunofluorescence and Histological Analyses

Tumor-bearing mice were randomly divided into three groups (n=5) that received NS, DOX + CDDP, or HA-DOX-CDDP micelles *via* injection. The mice were sacrificed 8 days after the final injection. Mouse body and tumor weights were recorded at 1–2-day intervals. Tumors, major organs, and lymph nodes were washed with NS, fixed in 4% paraformaldehyde, and embedded in paraffin. Tissues in paraffin blocks were cut into ~5-μm slices for hematoxylin and eosin (H&E) staining and ~3-μm slices for immunofluorescence (PARP and survivin) analyses. Alterations in pathological histology and fluorescence intensity were detected using a Nikon microscope (Eclipse Ti, Optical Apparatus Co., Ardmore, USA) and CLSM, respectively. All image data collected were analyzed using ImageJ software (National Institutes of Health, Bethesda, Maryland).

### Statistical Analysis

All experiments were performed in triplicate and results were analyzed for statistical significance using IBM SPSS 17.0 (IBM Company, Armonk, NY, USA). Differences between the groups were estimated using one-way ANOVA or t-tests, and reported as means ± standard deviations (SD). Two-tailed *P* values <0.05 were considered to indicate statistically significant results. When *P* values <0.01 or <0.001, results were considered to have statistically obvious difference.

## Results

### Physicochemical Properties of HA-DOX-CDDP

**Figure 1:**
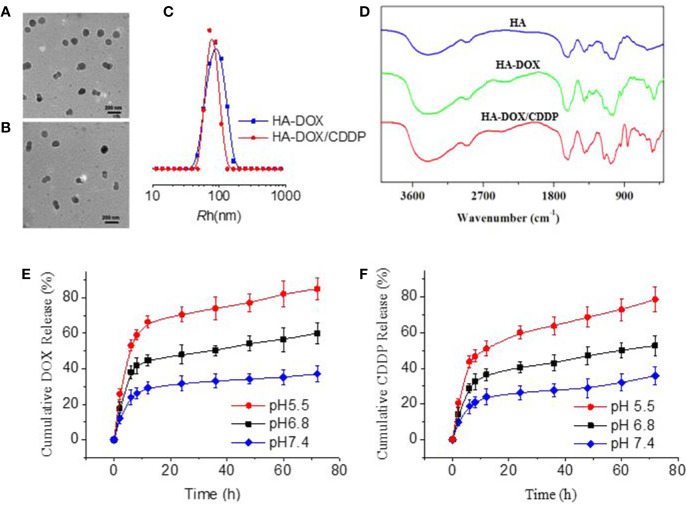
Characterization of DOX-loaded HA micelles. **(A–B)** Transmission electron microscopy images of **(A)** HA-DOX and **(B)** HA-DOX-CDDP. Scale bars: 200 nm. **(C)** Particle size of HA-DOX and HA-DOX-CDDP. **(D)** Fourier transform IR spectra of HA, HA-DOX, and HA-DOX-CDDP. **(E)** *In vitro* DOX release profiles of HA-DOX-CDDP across a range of pH values. **(F)** *In vitro* CDDP release profiles of HA-DOX-CDDP across a range of acidic pH values.

As shown in **Figure 1**, HA-DOX and HA-DOX-CDDP were spherical (**Figures 1A, B**). The diameters from TEM microimages were about 100 nm. The hydrodynamic radius (Rh) was about 80 nm. The relative smaller size from TEM detection should be attributed shrinking of nanoparticles during preparing the samples for TEM detection (**Figure 1C**). The FT-IR spectra of HA, HA-DOX, and HA-DOX-CDDP are shown in in **Figure 1D**. The absorption peaks of HA-DOX and HA-DOX-CDDP in the FT-IR spectra were 1300–1000 cm^-1^ duo to the stretching vibration of C-O bond in DOX, but in the spectra of HA-DOX-CDDP, the characteristic absorption peak at 3300–3200 cm^-1^ was also present duo to the amines (NH2) in CDDP. The waveforms of DOX-loaded HA micelles were nearly identical, and slightly different from those of HA, which indicated that the three types of micelles had similar chemical construction.

To determine the release characteristics of HA-DOX-CDDP under physiological conditions, and in the intracellular microenvironment, *in vitro* drug release of DOX and CDDP from micelles was evaluated at pH 5.5, 6.8, and 7.4 (**Figures 1E, F**). The micelles exhibited a burst release pattern during the first 4–6 h, and then showed sustained release at different acidic conditions. Doxorubicin and CDDP release from cross-linked micelles was less than 35% at pH 7.4. However, as pH decreased to 6.8 or 5.5, DOX and CDDP release from cross-linked micelles increased to approximately 50% and 80%, respectively, after 72 h.

### *In Vitro* Cellular Uptake

**Figure 2:**
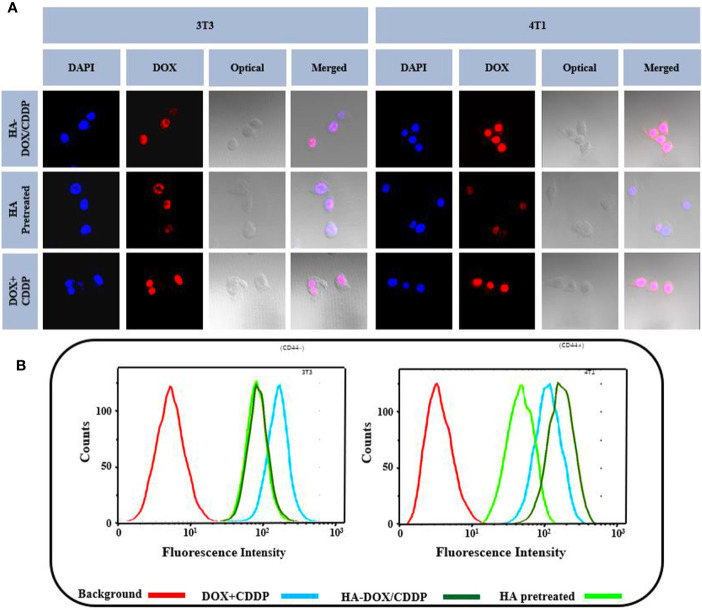
*In vitro* cellular uptake and intracellular DOX release in 3T3 and 4T1 cells. **(A)** Confocal laser scattering microscopy and **(B)** FCM analysis were performed on 3T3 and 4T1 cells following HA pretreatment, and treatment with free DOX+CDDP and HA-DOX-CDDP.

Flow cytometry and CLSM were used to investigate the internalization profiles of HA-DOX-CDDP micelles in 3T3 and 4T1 cells. After incubation with free drugs (DOX+CDDP) or HA-DOX-CDDP for 6 h, the highest levels of free drug internalization were observed in 3T3 cells. In CD44-positive 4T1 cells, HA-DOX-CDDP-treated groups exhibited significantly higher internalization than groups treated with free drugs or pretreated with HA (**Figure 2A**). In addition, the fluorescence intensity induced by HA-pretreatment was almost similar to that induced by HA-DOX-CDDP treatment in 3T3 cells, but less than that following treatment with free drugs in both 3T3 and 4T1 cells. The results obtained from FCM analysis were consistent with those obtained using CLSM (**Figure 2B**).

### Inhibition of Cell Proliferation

**Figure 3:**
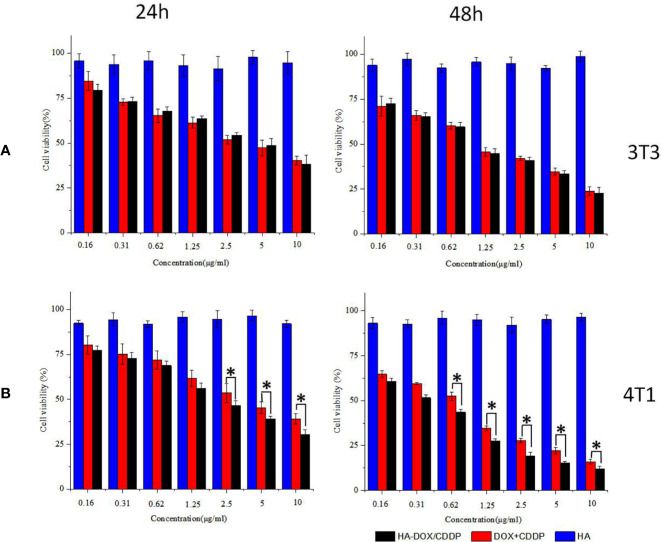
*In vitro* cytotoxicity assay. **(A)** Cell viability of 3T3 cells treated with HA, DOX+CDDP, and HA-DOX-CDDP for 24 or 48 h. **(B)** Cell viability of 4T1 cells treated with HA, DOX+CDDP, and HA-DOX-CDDP for 24 or 48 h. Data are presented as the mean ± SD (n = 3). *P < 0.05 compared with the DOX+ CDDP group.

Inhibition of cell proliferation in response to DOX-loaded HA micelles was evaluated using the MTT assay in 3T3 and 4T1 cells following 24 or 48 h of exposure (**Figure 3**). Groups treated with the free drugs (DOX+CDDP) and HA-DOX-CDDP micelles both exhibited time- and dose-dependent cytotoxicity. However, treatment with HA groups induced slightly smaller effects on cell viability, indicating low cytotoxicity against the two cell types. Compared with groups treated with free drugs, HA-DOX-CDDP-treated groups exhibited significantly more cytotoxicity toward 4T1 cells, which express high levels of CD44 receptors, following treatment at 2.5, 5, and 10 μg/ml for 24 h, or treatment at a concentration of 0.65 μg/ml or higher for 48 h (**Figure 3B**). In contrast, no significant differences in cell viability were observed following treatment with free drugs or HA-DOX-CDDP in 3T3 cells, which express low levels of CD44. HA-DOX-CDDP against the 3T3 and 4T1 cells with the IC50s of 3.07 and 1.10 μg/ml for 24 h exposure, but 2.04 and 0.34 μg/ml for 48 h exposure. Moreover, free DOX plus CDDP against the 3T3 and 4T1 cells with the IC50s of 3.45 and 1.16 μg/ml for 24 h exposure, but 3.58 and 0.56 μg/ml for 48 h exposure.

### Inhibition of Cell Proliferation and HA-DOX-CDDP Penetration in 3D Cell Culture

**Figure 4:**
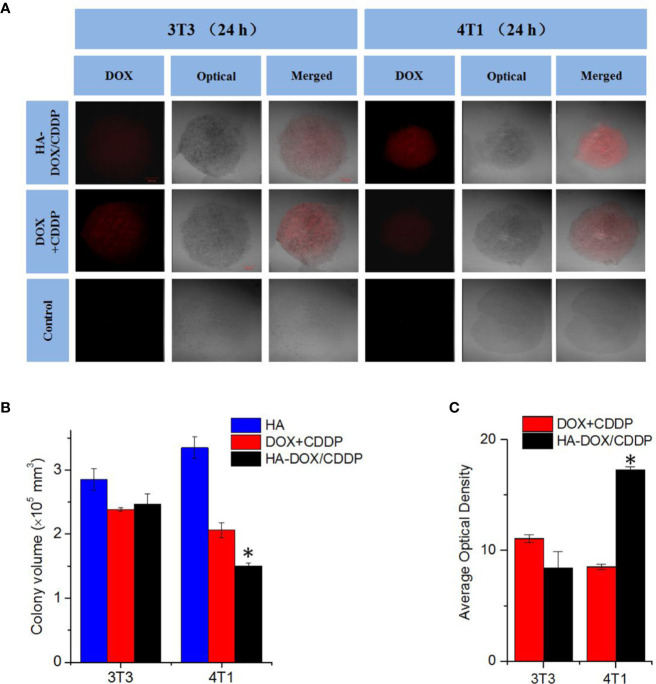
*In vitro* multicellular spheroids in 3D suspension cultures. **(A)** Confocal laser scattering microscopy of 4T1 and 3T3 cell spheroids treated with HA, DOX+ CDDP, and HA-DOX-CDDP for 24 h. **(B)** Colony volume and **(C)** fluorescence density analyses of 4T1 and 3T3 cell spheroids treated with HA, DOX+ CDDP, and HA-DOX-CDDP for 24 h. *P < 0.05 compared with the DOX+ CDDP group.

Three-dimensional multicellular spheroid models of 3T3 and 4T1 cells were established to further evaluate the tumor inhibition effects and cellular uptake of HA-DOX-CDDP micelles (see **Figure 4**). The 4T1 spheroid volumes in the HA-DOX-CDDP treatment group were significantly smaller than those in the free drugs group (*P* < 0.05). In contrast, no significant differences in 3T3 spheroid volume were observed between the HA-DOX-CDDP and DOX+CDDP groups (**Figures 4A, B**). In addition, drug penetration was evaluated following co-incubation with drugs for 24 h. Compared with the free drugs group, the cores of the multicellular 4T1 spheroids in the HA-DOX-CDDP group showed much stronger fluorescence intensity (*P*<0.05) (**Figures 4A, C**). Consistent with the proliferation results, no significant differences in fluorescence intensity were observed between the HA-DOX-CDDP and DOX+CDDP groups in the 3T3 cell model. These results demonstrate that the nanoparticulate drug formulation showed enhanced inhibition of cell proliferation and cellular uptake *via* CD44 receptors on the surfaces of 4T1 tumor cells.

### Drug Biodistribution *In Vivo*

**Figure 5:**
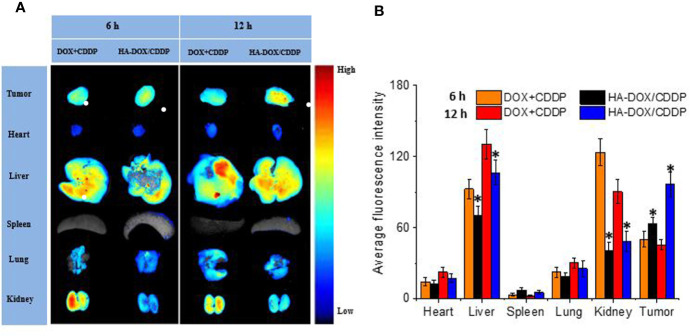
*In vivo* DOX biodistribution. **(A)** *Ex vivo* fluorescence images of isolated organs and tumors at 6 or 12 h post-injection. **(B)** Semi-quantitative analysis of the mean fluorescence intensity in isolated organs and tumors at 6 or 12 h post-injection. Data are presented as the mean ± SD (n=3). *P < 0.05 compared with the DOX+CDDP group.

Drug distribution in major organs and isolated tumors was evaluated 6 or 12 h after injection of free or nanoparticulate drugs (**Figure 5**). The livers and kidneys showed strong fluorescence in the HA-DOX-CDDP and free DOX+CDDP groups 6 h post-injection, and the fluorescence intensity was higher at 12 h (**Figure 5A**). These results may be attributed to the high rate of metabolism of small-molecule anticancer drugs in the liver and kidney (Wicki et al., 2015; Huang et al., 2017). The fluorescence signals in major organs were significantly weaker for the HA-DOX-CDDP group than for the free drug group at both 6 and 12 h post-injection, except in the spleen, suggesting lower levels of drug distribution in the spleen relative to the other organs and tissues. In contrast, the fluorescence intensity in tumors was 1.3- and 2.1-fold higher in the HA-DOX-CDDP group than in the free drug groups after 6 and 12 h exposure, respectively (**Figure 5B**).

### *In Vivo* Tumor Suppression

**Figure 6:**
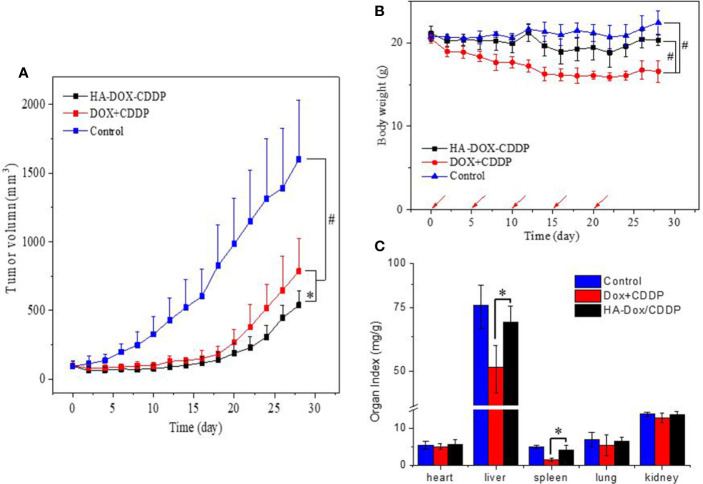
*In vivo* safety and antitumor efficacies. **(A)** Tumor volumes and **(B)** body weights of 4T1-xenografted mice after treatment with NS as the control, DOX +CDDP, or HA-DOX-CDDP. Red arrows showed the tail-veil injection time. **(C)** Organ coefficients of isolated organs in NS, DOX +CDDP, or HA-DOX-CDDP treated groups. *P < 0.05, ^#^*P* < 0.001.

The tumor suppression effect of NPs and free drugs *in vivo* was evaluated in BALB/c mice injected with 4T1 cells in the abdominal fat pad. Tumor volumes and body weights were calculated at the end of the experiment. As shown in **Figure 6A**, the tumors progressed rapidly with an average volume of 1600 ± 487 mm^3^ in the control group. In contrast, the tumors were significantly smaller in the HA-DOX-CDDP- and DOX+CDDP-treated groups. Treatment with HA-DOX-CDDP resulted in 66% tumor inhibition compared with that in the other treatment groups. In addition, body weight decreased rapidly in the free DOX+CDDP group during the initial 10 days of treatment, then stabilized gradually. Overall, the animals in this group experienced a 19% loss in body weight (**Figure 6B**). In the HA-DOX-CDDP group, body weight decreased at a rate similar to that in the control group during the early treatment stage, and total body weight loss was 3.7%. Body weight did not significantly change in the control group. As shown in **Figure 6C**, organ coefficients were calculated to evaluate side effects of drug treatment. In the free DOX+CDDP group, the organ coefficients of the liver and spleen were obviously lower than in the other two groups (*P*<0.01). There were no significant differences in organ coefficients between the HA-DOX-CDDP and control groups.

### Histopathology and Immunofluorescence Analyses

**Figure 7:**
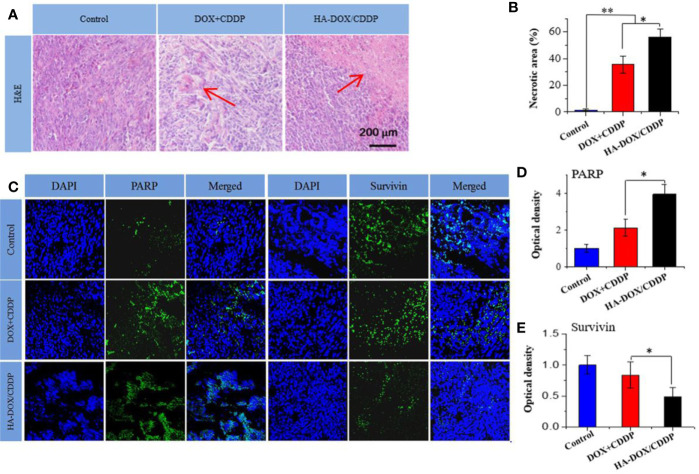
Histopathology and immunofluorescence analyses. **(A)** Histopathological (H&E) analyses and **(B)** necrotic areas in H&E-stained tumor sections from 4T1-xenografted mice following treatment with NS as the control, DOX+CDDP, or HA-DOX-CDDP. Red arrows indicated the necrotic area. **(C)** Immunohistochemical (PARP and survivin) analyses of tumor tissue sections following treatment with NS as the control, DOX +CDDP, or HA-DOX-CDDP. **(D)** Relative optical densities of tumor sections showing PARP immunofluorescence. **(E)** Relative optical densities of tumor sections showing survivin immunofluorescence. Data are presented as the mean ± SD (n = 5). *P < 0.05, **P < 0.01.

Pathological analyses of tumor tissues were performed to confirm the anticancer efficacies of the different drug formulations. As shown in **Figure 7A**, most tumor cells exhibited integrated cellular morphologies in the control group. In contrast, tumor cell necrosis was observed in the HA-DOX-CDDP and DOX+CDDP groups. Tissues in the HA-DOX-CDDP group had larger necrotic areas (56.3% ± 5.8%) than those in the free DOX+CDDP (35.65 ± 6.5%) and control groups (1.5% ± 0.8%) (**Figure 7B**).

To investigate mechanisms of apoptosis in tumor cells following treatment, immunofluorescence of PARP and survivin was evaluated in tumor tissue sections. As shown in **Figure 7C**, the fluorescence density of PARP in the control group was lower than that in the other two groups. Furthermore, PARP fluorescence intensity in the HA-DOX-CDDP group was approximately 4.15- and 2.23-fold higher than that in the free drugs and control groups, respectively (**Figure 7D**). Compared with the control group, the fluorescence intensity of survivin was lower, to differing extents, in the HA-DOX-CDDP and DOX + CDDP groups. Furthermore, the HA-DOX-CDDP groups showed significantly weaker survivin fluorescence than the free drugs group (**Figure 7E**).

## Discussion

Many attempts have been made to develop effective targeted anti-cancer drug delivery systems with improved safety and reduced side effects. Nanotechnology has enabled significant improvements in therapeutic efficacy (Zhou et al., 2015; Gupta et al., 2017; Youn and Bae, 2018). In the present study, an HA-modified nanoparticle was designed to encapsulate DOX and CDDP *via* side carboxyl (COOH) groups as a novel formulation for treatment of breast cancer. Doxorubicin, a chemotherapy drug that is widely used clinically, reduces DNA repair capacity, and has been shown to exert synergistic effects with CDDP for treatment of breast cancer (Roy et al., 2018; Guo et al., 2019) Previous studies used CDDP as a crosslinker for chelating HA and DOX at an optimized ratio to generate stable drug-loaded nanogels with suitable particle sizes (Harrington et al., 2000; Huang et al., 2018). The stability and pH-sensitivity of HA-DOX-CDDP were verified at pH 7.4, 6.8, and 5.5 in our study to simulate normal physiological microenvironments, acidic tumor tissue, and lysosomal microenvironments, respectively (Fan et al., 2015; Cheng et al., 2019). Extended incubation of HA-DOX-CDDP resulted in DOX release rates that increased as pH decreased from 7.4 to 5.5. This effect was likely attributable to the disruption of carboxyl groups between HA and DOX under acidic conditions. These results suggest that the pH-sensitive nature of HA-DOX-CDDP may result in more rapid drug release in the tumor microenvironment and sustained release under normal physiological conditions, which may lead to increased antitumor activity. In addition, HA-modified nanoparticles exhibited targeted delivery to cancer cells that expressed high levels of CD44. In this study, NIH-3T3 (CD44^-^) and 4T1 (CD44^+^) cell lines were used to evaluate the anticancer potential of the developed formulation against breast tumors. Doxorubicin fluorescence intensity following treatment with HA-DOX-CDDP was observed in the nucleus at the same intracellular location as that observed following treatment with free DOX. In addition, fluorescence was remarkably enhanced in 4T1 cells, but only slightly in NIH-3T3 cells, compared with that in the HA-pretreated or free drug groups. These results may have been due to specific internalization of HA-DOX-CDDP through interaction with CD44 receptors on the surface of 4T1 cells. These findings indicate that DOX-loaded HA micelles exhibit optimal CD44-mediated targeting ability and pH-dependent drug release in the acidic tumor microenvironment, which could result in enhanced breast tumor-targeting efficiency and reduced drug-induced side effects.

The effects of DOX-loaded HA micelles on proliferation and growth inhibition of NIH-3T3 and 4T1cells were further confirmed the security evaluation and selective toxicity for aforesaid drug delivery systems. *In vitro* cytotoxicity assay showed that HA treatment induced negligible cytotoxicity in both cell lines, at different concentrations, which indicated good biocompatibility. Furthermore, DOX-loaded HA micelles inhibited cell growth to a greater extent than free DOX+CDDP in 4T1cells, but not in 3T3 cells, after 72 h of treatment, indicating higher cytotoxicity and selectivity of HA-DOX-CDDP. These observations are likely attributable to HA receptor-mediated endocytosis and the delayed drug release characteristic of nanosized micelles. These results agreed with those obtained using a 3D spheroid culture model, which was more representative of cellular functions and physiological responses in tissues and organs (Perin et al., 2017).

Drug distribution in tissues has been associated with therapeutic efficacy and potential organ toxicity (Salimi et al., 2018). In this study, higher accumulation of free DOX was observed in the liver and kidney in the free DOX+CDDP-treated mice. This may have been due to substantial phagocytosis by liver macrophages and rapid renal metabolism of small molecule anticancer agents (Taurin et al., 2012; Guo et al., 2018; Ding et al., 2019). In contrast, HA-DOX-CDDP treatment resulted in increased accumulation of free DOX only in breast tumor tissues. Furthermore, free DOX concentrations were lower in other organs in HA-DOX-CDDP mice than those in the free drugs-treated mice, particularly in the kidney. These results may have been due to enhanced EPR effect, excellent biocompatibility, and specific targeting to CD44 receptors. Furthermore, treatment with HA-DOX-CDDP resulted in lower tumor volume and lower loss of body weight than treatment with free DOX+CDDP. These results showed that selective biodistribution of HA-DOX-CDDP resulted in DOX accumulation at breast tumor sites and reduced systemic toxicity following intravenous injection.

Histopathological and immunofluorescence studies were performed to evaluate drug efficacy and side effects following repeated intravenous administration. In our experiments, HA-DOX-CDDP treatment resulted in the greatest tumor necrotic areas among all of the groups, which indicated increased activity against breast cancer. Furthermore, the expression of PARP was increased and the expression of survivin was decreased in tumor sections of mice treated with HA-DOX-CDDP compared with those in mice treated with free DOX+CDDP. PARP is an important nuclear protein involved in DNA repair signaling pathways that can either maintain the structural integrality of chromosomes or mediate necrosis by inducing DNA damage (Pascal, 2018). Hyper-activation of PARP triggers DNA fragmentation and cell necrosis through enhanced release of apoptosis-inducing factor (Dale et al., 2015; Francica and Rottenberg, 2018). Survivin, an apoptosis inhibitor, plays a crucial role in tumor cell differentiation, progression, and invasion (Jaiswal et al., 2015). In particular, surviving expression has been shown to be an independent prognostic factor based on overexpression in neoplastic tissues and low expression in normal tissues (Veiga et al., 2019; Mahmoudian-Sani et al., 2019). The results of our study indicated that HA-DOX-CDDP showed greater therapeutic efficacy against breast cancer than free drugs.

In conclusion, the present CDDP-crosslinked DOX-loaded HA micelles prepared with innocuous methods and possessed an outstanding ability to control the release of DOX and CDDP for pH sensitivity and CD44 targeting.HA-DOX-CDDP exhibited synergistic anticancer effects, tumor-targeted ability, and reduced multi-organ toxicity compared with conventional anti-breast cancer agents. These results indicate that HA-DOX-CDDP may represent a multifunctional nano-drug delivery system that exhibits improved therapeutic efficacy against breast cancer. This study also provides avenues for exploring the incorporation of selective agents with biological targeting to overcome severe adverse effects in carcinoma chemotherapy.

## Data Availability Statement

All datasets generated for this study are included in the article/supplementary material.

## Ethics Statement

The animal study was reviewed and approved by the Institutional Animal Care and Use Committee of Jilin University.

## Author Contributions

QL conceived and designed the experiments. TY and YL contributed significantly to cell culture and drug treatments, data compilation and analysis, and manuscript preparation. XG performed the mice treatments and HA-DOX-CDDP preparation.

## Conflict of Interest

The authors declare that the research was conducted in the absence of any commercial or financial relationships that could be construed as a potential conflict of interest.
